# Does China’s Pilot Carbon Market Cause Carbon Leakage? New Evidence from the Chemical, Building Material, and Metal Industries

**DOI:** 10.3390/ijerph20031853

**Published:** 2023-01-19

**Authors:** Jianhui Cong, Huimin Wang, Xiaoxiao Hu, Yongbin Zhao, Yingying Wang, Weiqiang Zhang, Ling Zhang

**Affiliations:** 1 School of Economics and Management, Shanxi University, Taiyuan 030006, China; 2 The Center for Economic Research, Shandong University, Jinan 250100, China; 3 School of Economics and Management, North University of China, Taiyuan 030051, China; 4 School of Economics and Management, Shanxi Normal University, Taiyuan 030031, China; 5 Institute of Blue and Green Development, Shandong University, Weihai 264209, China; 6 School of Business and Economics, Free University of Berlin, 14195 Berlin, Germany; 7 School of Economics and Management, China University of Petroleum (Beijing), Beijing 102249, China

**Keywords:** carbon market, carbon leakage, input–output, difference-in-differences

## Abstract

The carbon market is an effective market for reducing greenhouse gas emissions; however, the existence of carbon leakage affects the emissions reduction effect of the carbon market. Using the multiregional input–output (MRIO) model and the difference-in-differences (DID) methodology, this study examined whether the chemical, building materials, or metals industries in China’s pilot carbon market have caused carbon leakage, the extent of the carbon leakage, and the areas to which the industries with carbon leakage have transferred their carbon emissions. The results showed that the pilot carbon market caused carbon leakage in the chemical, building materials, and metal industries. The building materials industry had the most serious carbon leakage, followed by the chemical industry, and the metal industry was the weakest. In addition, regardless of the industry, most of the areas affected by carbon leakage were concentrated in regions with relatively backward economic development and weak in-place environmental regulations, such as in the central and western regions. Compared with the other pilot areas, Guangdong was the area most likely to be affected by carbon leakage from other pilot areas. This study provides new evidence for the existence of carbon leakage in China’s pilot carbon market from an industrial perspective.

## 1. Introduction

The carbon market is an important tool for managing climate change and reducing greenhouse gas emissions. In 2005, the EU took the lead in launching the European Union emissions trading scheme (EU ETS) to reduce greenhouse gas emissions. At the beginning of 2022, there were 25 carbon markets in operation worldwide [1]. In October 2011, China approved the implementation of carbon-emissions-trading pilot projects in seven areas, including Beijing, Tianjin, Shanghai, Chongqing, Hubei, Guangdong, and Shenzhen, covering nearly 3000 key emissions units in more than 20 industries such as power, steel, and cement. Many scholars have shown that the establishment of the carbon market has effectively promoted China’s emissions reductions [2,3,4]. China’s carbon market began online trading in 2021. According to data from the Ministry of Ecology and Environment, the national carbon market includes the first batch of 2162 key emissions units in the power generation industry, covering approximately 4.5 billion tons of carbon dioxide emissions [5]. The steel, non-ferrous metals, petrochemical, chemical, building material, papermaking, power, and aviation industries should gradually be incorporated into the carbon market. In September 2021, China unveiled a guiding document on the country’s efforts to achieve its carbon peak and neutrality goals under the new development philosophy, which specifically mentioned the need to accelerate the construction and improvement of the national carbon market. The carbon market is an important policy for China to achieve its dual carbon goals (China will strive to reach peak carbon dioxide emissions before 2030 and achieve carbon neutrality before 2060).

An imbalance in environmental policies causes carbon leakage, thus affecting the emissions reduction effect [6,7]. The existence of carbon leakage has always been a focus of policy communities. To effectively manage carbon leakage, the EU has carried out a carbon leakage risk assessment for various industries and proposed detailed anti-carbon leakage policies in the revised national assistance guide for the carbon-emissions-trading system of the EU. As the largest carbon market in the world, China’s carbon market has not yet introduced specific anti-carbon leakage measures. The presence or absence of policies between pilot and non-pilot areas in China and the difference in policy intensity between the pilot areas have led to an imbalance in emissions reduction policies among different areas; in theory, there is a possibility of carbon leakage. After further expansion of the industry coverage of China’s pilot carbon market, the existence of carbon leakage could directly affects the market competitiveness of the relevant emissions control industries. Therefore, studying the existence of carbon leakage in China’s pilot carbon market is of great significance for the construction and improvement of China’s carbon market, the realization of China’s dual carbon goal, and its responses to climate change.

As the first organization to establish an emissions trading system, many scholars have conducted studies on carbon leakage in the EU, especially on carbon leakage from the EU to China as well as on carbon leakage at the industry level in the EU. A small number of studies have also analyzed carbon leakage in China’s pilot carbon market, creating a new perspective for the study of China’s carbon market. However, few studies have focused on carbon leakage at the industry level or a summary of the specific path of carbon leakage, which limits its contribution to the formulation of China’s anti-carbon-leakage policy.

This study aimed to evaluate carbon leakage in the pilot carbon market at the industry level, explore the specific carbon leakage path, and propose targeted anti-carbon-leakage policies, thereby providing a reference for the construction and improvement of China’s carbon market. This paper combined the MRIO and DID models to examine whether the pilot carbon market has caused carbon leakage in the chemical, building materials, and metal industries. We use the MRIO model to analyze the areas where industries with carbon leakage were most likely to leak carbon emissions. Finally, combined with our results and based on the EU’s anti-carbon-leakage policy, this study provided valuable suggestions for China’s carbon market to effectively manage carbon leakage.

The remainder of this paper is organized as follows. Section 2 reviews the relevant literature on carbon leakage, and Section 3 introduces the theoretical mechanism of carbon leakage. Section 4 introduces the methods and data used in this study. Section 5 introduces the empirical analysis results of the DID model and carbon leakage path. Section 6 presents an analysis and discussion of the results. Finally, we draw conclusions and propose policy suggestions.

## 2. Literature Review

Since the signing of the United Nations Framework Convention on Climate Change, the issue of carbon leakage has aroused extensive discussion in academic circles. The Intergovernmental Panel on Climate Change (IPCC) pointed out that carbon leakage refers to the emissions reduction policies of Annex I countries in the Kyoto Protocol, which may lead to an increase in the carbon emissions of countries without emissions reduction constraints to offset some or even all of the emissions reductions. Generally, this implies that emissions reduction policies in abating areas have led to an increase in carbon emissions in non-abating areas [7,8]. Strictly speaking, carbon leakage can be divided into strong and weak categories. The IPCC defines carbon leakage as strong carbon leakage, i.e., carbon transfer caused by unbalanced emissions reduction policies [7]. Weak carbon leakage has a broader meaning. It is caused by various factors, such as factor endowment, production technology, and climate policy. Trade is the direct medium [8,9]. This study mainly focused on strong carbon leakage.

Many scholars have suggested that there are three main channels of strong carbon leakage between emissions reduction and non-emission-reduction countries: trade in energy products, trade in carbon-intensive products, and the transfer of energy-intensive industries [7,10,11,12,13]. The trade channel of energy products refers to the reduction in fossil fuel demand in abating areas due to emissions reduction targets, resulting in a reduction in fossil fuel prices in the energy market. This will increase the purchase and use of fossil fuels in non-abating areas, resulting in an increase in carbon emissions in non-abating areas. The trade channel of carbon-intensive products refers to the fact that the production cost of carbon-intensive products in abating areas may rise due to emission reduction constraints, so as to weaken the competitiveness of products in abating areas in the market and indirectly enhance the market competitiveness of products in areas without emission reduction constraints. This will promote more market demand to non-abating areas, resulting in an increase in carbon emissions in non-abating areas. The last carbon leakage channel refers to that due to the rise of production costs, enterprises in abating areas are more inclined to transfer industries or investment to areas without emission reduction constraints or relatively loose emission reduction policies in order to obtain greater profits, resulting in the increase in carbon emissions in non-abating areas.

Analyzing carbon leakage requires further discussions on the attribution of carbon emissions responsibility. The Kyoto Protocol provided a measurement of emissions from the producers’ perspective. However, in the context of an increasing proximity of global economic ties, producers are not the only consumers of their products [14]. Carbon emissions accounting based only on production principles is considered one of the causes of carbon leakage [15,16,17]. Therefore, many scholars have proposed the calculation of carbon emissions based on consumption. Rocco et al. suggested that carbon emissions accounting based on the consumption principle could effectively alleviate carbon leakage caused by accounting based on production principles [15]. Yu et al. found that carbon emissions accounting based on the consumption principle can significantly reduce the carbon leakage rate by 4% through a summary and analysis of previous studies [9]. Jakob suggested that both producers and consumers benefit from economic activities that produce emissions [18]. Therefore, neither the producers nor consumers can suitably bear the responsibility for carbon emissions alone and should bear it together at a certain proportion.

Regarding the calculation of carbon leakage, previous research has been divided into ex ante and ex post analyses. Ex ante analysis mainly uses the Computable General Equilibrium (CGE) model to calculate the carbon leakage rate. The carbon leakage rate is a common academic measure of the extent of carbon leakage. This is derived from the ratio of the increase in emissions in non-abating areas caused by abatement policies to the reduction in emissions in abatement areas [19]. As the carbon leakage rate is sensitive to the assumptions of the model, the carbon leakage rate calculated by different studies using the equilibrium model varies significantly [18,20,21]. The ex post analysis mainly adopts an econometric model: the most involved econometric model is the difference-in-differences model. To calculate weak carbon leakage, scholars usually use a multiregional input–output model [9]. This study used both the MRIO and DID models, combining their advantages, and fully analyzed the carbon leakage in China.

Presently, research on carbon leakage has mostly focused on carbon leakage in the EU ETS. As the first organization to establish an emissions-trading system, many scholars have assessed the carbon leakage of EU ETS at the industry level [10,13,22,23,24,25,26]. Demailly and Quirion [23] and Ponssard and Walker [26] estimated the carbon leakage rates in the steel and cement industries, respectively. Zhou and Sheng [13] analyzed the imports and exports of EU-based cement, aluminum, and steel industries using a structural breakpoint test, but they only found some evidence of carbon leakage in the import of steel. Paroussos et al. [10] found that the total carbon leakage rate of the EU from 2015–2050 was approximately 28%; it was found that the chemical and metal industries were the industries prone to high leakage rates. Boutabba and Lardic [22] used the rolling cointegration method to examine whether and to what extent the cement and steel industries were affected by carbon leakage. The results showed that these two industries were affected by negligible carbon leakage and loss of competitiveness. Additionally, Naegele and Zaklan [25] did not find any carbon leakage from European manufacturing.

To manage carbon leakage caused by differences in climate policies, the EU has proposed imposing carbon tariffs on carbon-intensive products in the EU imported from China [12,14]. However, whether the EU ETS caused carbon leakage from the EU to China has not yet been determined. Some scholars have found no evidence of carbon leakage from the EU to China. Zhao et al. [12] analyzed the trend in the import and export ratio of carbon-intensive products between China and the EU from 1992 to 2008, suggesting that there may be no carbon leakage between China and the EU. However, the study did not exclude the interference of factors other than the policy, which could not fully prove strong carbon leakage. Fu and Zhang [27] and Di [14] found carbon leakage from the EU to China. Fu and Zhang found carbon leakage in high-carbon manufacturing industries using the feasible generalized least squares (FGLS) method [27]. Di showed that the scale of carbon leakage from the EU to China generally had an upward trend from 1995 to 2011 [14]; their research mainly focused on weak carbon leakages. Additionally, whether carbon tariffs can effectively solve the problem of carbon leakage has aroused extensive discussion among scholars. Many studies have found that imposing carbon tariffs on developing countries is not an effective measure to alleviate carbon leakage, rather it will increase the carbon leakage rate, which is not conducive to the realization of the overall global emissions reduction target [28,29].

In addition to the EU’s carbon leakage, whether China’s pilot carbon market has caused strong carbon leakage has also become a hot research topic. Lin et al. used a partial equilibrium model to predict carbon leakage in the iron and steel industries under China’s carbon trading policy [30]. They found that the change in the carbon leakage rate of the industry was relatively stable. Gao, Yu et al., and Cao et al. conducted a post-evaluation study [6,9,31]. Gao et al. concluded that the pilot carbon market has led to carbon leakage from pilot to non-pilot areas by comparing the impact of the pilot carbon market on carbon emissions from the production and consumption sides [6]. However, their research investigated the average effect of industry and did not specifically analyze which industries had carbon leakage. Yu et al. used the propensity-score-matching and differences-in-differences (PSM-DID) method to evaluate the impact of the pilot carbon market on China’s foreign direct investment decision-making, finding that China’s carbon market promoted carbon leakage through investment channels [9]. Cao et al. used power-plant-level data and found no evidence of carbon leakage from the power industry to other provinces in the pilot area [31]. Zhou et al. found reverse carbon leakage, i.e., carbon leakage from non-pilot to pilot areas [21]. Generally, there have been few studies on carbon leakage in China’s carbon market, especially in specific industries.

In summary, the current research on carbon leakage mostly focuses on carbon leakage in carbon-intensive industries in the EU ETS and carbon leakage from the EU to China. Some scholars have examined the weak carbon leakage between Chinese provinces and the strong carbon leakage caused by the pilot carbon market. However, there have been few studies on strong carbon leakage in specific industries in the pilot carbon market; there is a lack of a summary of carbon leakage paths in industries with carbon leakage. With the development of the national carbon market, the formulation of targeted anti-carbon-leakage policies is an urgent problem. Compared with previous studies, the marginal contributions of this study were as follows. (1) The existence of carbon leakage in three typical industries of the pilot carbon market (chemical, building materials, and metal industries) was assessed at the industry level, and the degree of carbon leakage in these three industries was compared. (2) The path of carbon leakage from pilot to non-pilot areas and between pilot areas was summarized in detail and the areas that were most likely to transfer carbon emissions from industries with carbon leakage in the pilot areas was analyzed, thus providing a reference for other regions to avoid carbon leakage.

## 3. Theoretical Mechanism

**Figure 1:**
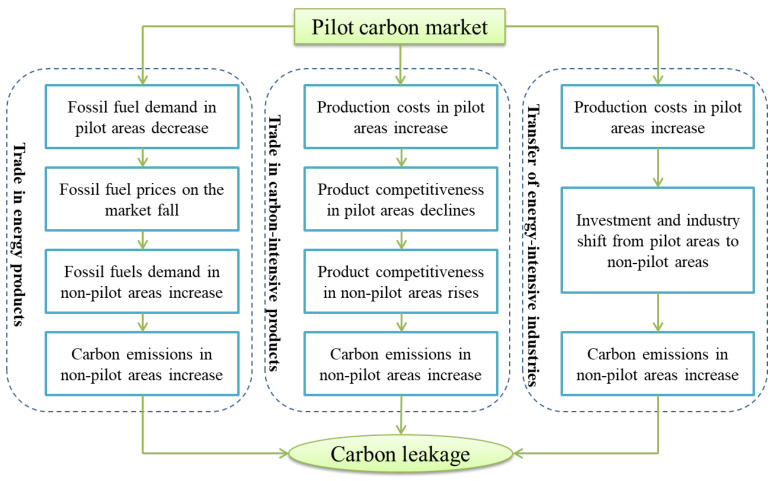
Theoretical mechanisms of carbon leakage.

The trade in energy products, the trade of carbon-intensive products, and the transfer of energy-intensive industries are also applicable to the chemical, building material, and metal industries in pilot and non-pilot areas (as shown in Figure 1). The chemical, building materials, and metal industries are carbon-intensive. Enterprises in the pilot areas may reduce the demand for fossil fuels due to emission reduction targets, resulting in lower prices of fossil fuels. Non-pilot areas may increase the demand for fossil fuels, leading to an increase in carbon emissions in non-pilot areas, resulting in carbon leakage through the trade channels of energy products. Additionally, the implementation of the pilot carbon trading policy will lead to an increase in the cost of emissions reduction enterprises in the pilot area. On the one hand, enterprises participating in the carbon market increase the cost of obtaining quotas. In addition to free quotas, enterprises must obtain quotas through auctions. If an enterprise’s carbon emissions exceed the quota, the excess component requires the enterprise to purchase the quota; otherwise, it will be punished. On the other hand, enterprises participating in the carbon market increase the cost of emissions reduction; they must reduce carbon emissions through process and technological innovations. Due to the increase in production costs, the competitiveness of the products in the pilot areas may be reduced, which indirectly enhances the competitiveness of the products in the non-pilot areas and urges the market demand to shift more toward the non-pilot areas. This will lead to an increase in carbon emissions in non-pilot areas and eventually lead to carbon leakage in pilot areas through the trade channels of carbon-intensive products. In addition, enterprises in the pilot areas will be more inclined to transfer industries or investments to non-pilot areas due to the increase in production costs, resulting in carbon leakage through the transfer channel of energy-intensive industries. To achieve the emissions reduction target, enterprises reduce emissions from a producer perspective while ignoring their emissions reduction responsibilities as consumers. This results in an imbalance of emission reductions on the production and consumption sides in the pilot area, which allows the market demand to shift more from the pilot area to the non-pilot area, resulting in a reduction in carbon emissions in the pilot area and an increase in carbon emissions in the non-pilot area. This study calculated carbon leakage based on this perspective and evaluated whether the pilot carbon market has caused strong carbon leakage by comparing the differences in carbon emissions reductions on the production and consumption sides in the pilot areas.

We note that this study focused on domestic carbon leakage without considering carbon leakage from pilot areas to foreign countries. On the one hand, this paper mainly calculated carbon leakage from an embodied carbon emissions perspective. The trade level between domestic provinces is significantly greater than that between domestic provinces and other countries; the scale of the carbon transfer between domestic provinces is significantly greater than that caused by foreign trade [32]. On the other hand, due to the limitations of China’s multi-regional input–output table, it was impossible to distinguish the countries with and without carbon markets in the input–output table. Therefore, this study did not consider carbon leakage from pilot areas to foreign countries.

## 4. Methodology and Data

This study combined the MRIO and DID models to determine whether there was strong carbon leakage in the chemical, building materials, and metal industries in China’s pilot carbon market. First, we used the MRIO model to calculate the production- and consumption-based carbon emissions of the chemical, building materials, and metals industries in various provinces in 2007, 2010, 2012, 2015, and 2017. Based on these data, the DID model was further applied to assess the impact of the pilot carbon market on the carbon emissions from the production and consumption sides of the three major industries in the pilot area to further derive the carbon leakage situation (the difference between the emissions reduction effect of the production and consumption sides).

Additionally, this study used the MRIO model to calculate the embodied carbon transfer between pilot and non-pilot areas and between pilot areas. By comparing the dynamic changes in carbon transfer before and after the implementation of the pilot carbon trading policy, this study further analyzed the areas where the industries with carbon leakage in the pilot areas were most likely to transfer carbon emissions.

### 4.1. Carbon Emissions of Production- and Consumption-Based and Transfer in and out

The MRIO model is an effective tool for measuring consumption-based carbon emissions and embodied carbon transfers [17,33]. The multiregional input–output table effectively connects the complex economic links between various regions and sectors, which is the empirical basis of the MRIO model. Assuming that there are *m* different regions and *n* different sectors in the table, the row-balance relationship is as follows:

(1) $$Q_{i}^{s}=\sum_{r=1}^{m}\sum_{j=1}^{n}X_{ij}^{sr}+\sum_{r=1}^{m}Y_{i}^{sr}+EX_{i}^{s}$$

Equation (1) shows that the total output ($Q_{i}^{s}$) is equal to intermediate use ($\overset{m}{\underset{r=1}{\sum }}\overset{n}{\underset{j=1}{\sum }}X_{ij}^{sr}$) and final use ($\overset{m}{\underset{r=i}{\sum }}Y_{i}^{sr}$(final consumption) + $EX_{i}^{s}$(export)), where $Q_{i}^{s}$ represents the total output of sector *i* in region *s*; $X_{ij}^{sr}$ refers to the products provided by department *i* in region *s* when department *j* in region *r* produces; $Y_{i}^{sr}$ refers to the final product produced by sector *i* in region *s*, which is finally used by region *r*; and $EX_{i}^{s}$ represents the final product produced by sector *i* in region *s* for export.

We introduced the direct consumption coefficient, $a_{ij}^{sr}$(*i,j* = 1…*n*; *s,r* = 1…*m*), which represents the direct input of department *i* in region *s* required by the unit output of department *j* in region *r*:

(2) $$a_{ij}^{sr}=X_{ij}^{sr}/Q_{j}^{r}$$

We introduced the direct consumption coefficient into Equation (1):

(3) $$Q_{i}^{s}=\sum_{r=1}^{m}\sum_{j=1}^{n}a_{ij}^{sr}Q_{j}^{r}+\sum_{r=1}^{m}Y_{i}^{sr}+EX_{i}^{s}$$

We then converted Equation (3) into matrix form:

(4) Q=AQ+Y+EX

(5) $$Q={\left(I-A\right)}^{-1}(Y+EX)=B(Y+EX)$$

(6) $$B=\left[\begin{array}{ccccc} b_{11}^{sr} & \cdots & b_{1j}^{sr} & \cdots & b_{1n}^{sr}\\ \vdots & \vdots & \vdots & \vdots & \vdots \\ b_{i1}^{sr} & \cdots & b_{ij}^{sr} & \cdots & b_{in}^{sr}\\ \vdots & \vdots & \vdots & \vdots & \vdots \\ b_{n1}^{sr} & \cdots & b_{nj}^{sr} & \cdots & b_{nn}^{sr} \end{array}\right]$$

where *Q* is the total output column vector; *A* is the direct consumption coefficient matrix; *Y* is the final consumption column vector; *EX* is the export column vector; *I* is the unit matrix; $B={\left(I-A\right)}^{-1}$ is the Leontief inverse matrix; and $b_{ij}^{sr}$ is the complete consumption coefficient.

As this study focused on carbon leakage in domestic pilot and non-pilot areas, the export situation was not considered. The MRIO model can be expressed as follows:

(7) $$\left[\begin{array}{ccc} q_{1}^{11} & \cdot \cdot \cdot & q_{1}^{1m}\\ \vdots & \vdots & \vdots \\ q_{n}^{11} & \cdot \cdot \cdot & q_{n}^{1m}\\ \vdots & \vdots & \vdots \\ q_{1}^{m1} & \cdot \cdot \cdot & q_{1}^{mm}\\ \vdots & \vdots & \vdots \\ q_{n}^{m1} & \cdot \cdot \cdot & q_{n}^{mm} \end{array}\right]=\left[\begin{array}{ccccccc} b_{11}^{11} & \cdots & b_{1n}^{11} & \cdots & b_{11}^{1m} & \cdots & b_{1n}^{1m}\\ \vdots & \vdots & \vdots & \vdots & \vdots & \vdots & \vdots \\ b_{n1}^{11} & \cdots & b_{nn}^{11} & \cdots & b_{n1}^{1m} & \cdots & b_{nn}^{1m}\\ \vdots & \vdots & \vdots & \vdots & \vdots & \vdots & \vdots \\ b_{11}^{m1} & \cdots & b_{1n}^{m1} & \cdots & b_{11}^{mm} & \cdots & b_{1n}^{mm}\\ \vdots & \vdots & \vdots & \vdots & \vdots & \vdots & \vdots \\ b_{n1}^{m1} & \cdots & b_{nn}^{m1} & \cdots & b_{n1}^{mm} & \cdots & b_{nn}^{mm} \end{array}\right]\left[\begin{array}{ccc} y_{1}^{11} & \cdot \cdot \cdot & y_{1}^{1m}\\ \vdots & \vdots & \vdots \\ y_{n}^{11} & \cdot \cdot \cdot & y_{n}^{1m}\\ \vdots & \vdots & \vdots \\ y_{1}^{m1} & \cdot \cdot \cdot & y_{1}^{mm}\\ \vdots & \vdots & \vdots \\ y_{n}^{m1} & \cdot \cdot \cdot & y_{n}^{mm} \end{array}\right]$$

Embodied carbon emissions refer to all carbon emissions generated during the production of products and services embodied in goods [34]. A carbon emissions coefficient, $e_{i}^{s}$, was introduced, which was derived from the ratio of carbon emissions to the total output of sector *i* in region *s*. *Es* is the diagonal matrix of $e_{i}^{s}$. We established the relationship between carbon emissions and the multiregional input–output model.

Equation (7) decomposes the total output of each region into outputs that meet the final demand of the region and other regions. By multiplying the diagonal matrix of *Es* by the total output decomposition matrix, we can obtain the decomposition matrix of production-based carbon emissions [35].

(8) $$\left[\begin{array}{ccccccc} e_{1}^{1} & \cdots & 0 & \cdots & 0 & \cdots & 0\\ \vdots & \vdots & \vdots & \vdots & \vdots & \vdots & \vdots \\ 0 & \cdots & e_{n}^{1} & \cdots & 0 & \cdots & 0\\ \vdots & \vdots & \vdots & \vdots & \vdots & \vdots & \vdots \\ 0 & \cdots & 0 & \cdots & e_{1}^{m} & \cdots & 0\\ \vdots & \vdots & \vdots & \vdots & \vdots & \vdots & \vdots \\ 0 & \cdots & 0 & \cdots & 0 & \cdots & e_{n}^{m} \end{array}\right]\left[\begin{array}{ccc} q_{1}^{11} & \cdot \cdot \cdot & q_{1}^{1m}\\ \vdots & \vdots & \vdots \\ q_{n}^{11} & \cdot \cdot \cdot & q_{n}^{1m}\\ \vdots & \vdots & \vdots \\ q_{1}^{m1} & \cdot \cdot \cdot & q_{1}^{mm}\\ \vdots & \vdots & \vdots \\ q_{n}^{m1} & \cdot \cdot \cdot & q_{n}^{mm} \end{array}\right]=\left[\begin{array}{ccc} f_{1}^{11} & \cdot \cdot \cdot & f_{1}^{1m}\\ \vdots & \vdots & \vdots \\ f_{n}^{11} & \cdot \cdot \cdot & f_{n}^{1m}\\ \vdots & \vdots & \vdots \\ f_{1}^{m1} & \cdot \cdot \cdot & f_{1}^{mm}\\ \vdots & \vdots & \vdots \\ f_{n}^{m1} & \cdot \cdot \cdot & f_{n}^{mm} \end{array}\right]$$

The production-based carbon emissions of a region refer to the carbon emissions caused by meeting the final demand of the region and other regions. Therefore, the production-based carbon emissions of sector *i* in region *s* ($F_{p}^{s}$) can be expressed as follows ($f_{i}^{sr}$ represents the carbon emission transfer from sector *i* in region *r* to region *s*):

(9) $$F_{p}^{s}=\sum_{r}^{m}f_{i}^{sr}$$

The consumption-based carbon emissions of a region refer to the carbon emissions caused by the region and other regions to meet the final demand of the region. Therefore, the consumption-based carbon emissions of sector *i* in region *s* ($F_{c}^{s}$) can be expressed as follows ($f_{i}^{rs}$ represents the carbon emission transfer from sector *i* in region *s* to region *r*):

(10) $$F_{c}^{s}=\sum_{r}^{m}f_{i}^{rs}$$

Carbon emissions transfer in of one region refers to the carbon emissions caused by the region to meet the final demand of other regions. Therefore, the carbon emission transfer in of sector *i* in region *s* ($F_{in}^{s}$) can be expressed as follows:

(11) $$F_{in}^{s}=\sum_{r\neq s}^{m}f_{i}^{sr}$$

The carbon emissions transfer out of one region refers to the carbon emissions caused by other regions to meet the final demand of the region. Therefore, the carbon emission transfer out of sector *i* in region *s* ($F_{out}^{s}$) can be expressed as follows:

(12) $$F_{out}^{s}=\sum_{r\neq s}^{m}f_{i}^{rs}$$

The production-based carbon emissions, consumption-based carbon emissions, and carbon emissions transfer in and out calculated in this section provided the basis for the strong carbon leakage analysis and a carbon leakage pathway summary.

### 4.2. Difference-in-Differences Model

This study used the DID model to investigate whether the pilot carbon market has caused carbon leakage, i.e., whether the pilot carbon trading policy has caused an increase in carbon transfer from pilot to non-pilot areas, thus outsourcing carbon emissions to non-pilot areas. In 2013 and 2014, Beijing, Tianjin, Shanghai, Chongqing, Hubei, and Guangdong successively implemented carbon trading policies. (Shenzhen was not considered due to data limitations. Both Sichuan and Fujian were listed as pilot areas of carbon emissions trading in December 2016, which were not included in this paper.) However, in October 2011, the National Development and Reform Commission issued a notice on establishing a pilot carbon market. Enterprises in the pilot areas may prepare for emissions reduction in advance, which will have an impact on production activities and carbon emissions. Therefore, this study regarded 2012 as the year of policy implementation. Six pilot areas (The pilot carbon market in Tianjin does not include the non-metallic mineral products industry [36]. In this paper, the non-metallic mineral products industry was regarded as the building materials industry. Therefore, when examining the building materials industry, Tianjin was regarded as a non-pilot area.) were treated as treatment groups, and the other twenty-four areas (due to data limitations, Tibet, Hong Kong, Macao, and Taiwan were excluded) were treated as control groups. The changes in the control groups before and after policy implementation could be regarded as a pure time effect. By comparing the differences between the treatment and control groups before and after the implementation of the carbon market, we evaluated the impact of the pilot carbon trading policy.

In this study, the dependent variable of the regression model was carbon emissions, i.e., the production- and consumption-based carbon emissions of three industries in thirty provinces of China. The control variables included the main factors affecting carbon emissions [2,6,36,37]: population, economic development level, urbanization rate, economic export-oriented level, industrial structure, energy structure, technical level, environmental regulation intensity, per capita initial industry input, and per capita total industry output. The DID model was constructed as follows:

(13) $$y_{pit}=\beta_{0}+\beta_{1}Treat_{it}+\beta_{2}Post_{it}+\beta_{3}Treat_{it}\times Post_{it}+\sum \beta_{j}X_{it}+\mu_{i}+\gamma_{t}+\varepsilon_{it}$$

(14) $$y_{cit}=\alpha_{0}+\alpha_{1}Treat_{it}+\alpha_{2}Post_{it}+\alpha_{3}Treat_{it}\times Post_{it}+\sum \alpha_{j}X_{it}+\mu_{i}+\gamma +\varepsilon_{it}$$

where $y_{pit}$ refers to the production-based carbon emissions of region *i* in year *t*;$\;y_{cit}\;$ refers to the consumption-based carbon emissions of region *i* in year *t*; and *Treat_it_* is a dummy variable indicating whether the carbon market is carried out in region *i*. If the region was a pilot region, then the value was 1; otherwise, it was 0. *Post_it_* represents a time dummy variable, with a value of zero before 2012 and one after 2012. *X_it_* is a series of control variables in region *i*. Here, $\mu_{i}$ is the individual-fixed effect; $\gamma_{t}$ is the time-fixed effect; $\varepsilon_{\mathrm{it}}\;$is the random interference term; $\beta_{3}$ measures the effect of carbon emissions reduction on the production side; and $\alpha_{3}$ measures the effect of carbon emissions reduction on the consumption side. The difference between $\beta_{3}\;$and $\alpha_{3}$ reflects whether the pilot area caused carbon leakage. Additionally, this study used the formula ((production-based emissions reduction effect − consumption-based emissions reduction effect)/production-based emissions reduction effect) to measure the degree of carbon leakage in the pilot carbon market.

### 4.3. Data

#### 4.3.1. Research Industry Selection

This study examined three industries: chemical, building materials, and metal. The main reasons for this selection were as follows. (1) The chemical, building materials, and metal industries are mainly involved in the pilot carbon market. (2) These three industries are carbon-intensive, with large direct or indirect carbon emissions involved in their production processes. Thus, they have a high risk of carbon leakage. (3) These three industries are involved in the industries identified by the European Commission, which are considered to be at risk for carbon leakage from 2021–2030, as well as being industries identified by the National Assistance Guidelines, which are at risk for serious carbon leakage due to indirect emissions costs from 2021–2030. (4) China is implementing the construction of the national carbon market; therefore, the chemical, building materials, and metal industries will be included in the future. Thus, examining the carbon leakage of these three industries is of great significance for the construction of the national carbon market and the formulation of anti-carbon leakage policies.

According to the definition of the building materials and metal industries, this study analyzed the non-metallic mineral products industry and metal smelting and calendaring products industry in the input–output table according to the National Economic Industrial Classification (GB/T4754-2017). Based on the existing literature [38,39], this study used the non-metallic mineral products industry in the input–output table to represent the building materials industry and the metal smelting and calendaring products industry to represent the metal (steel and non-ferrous metals) industry.

#### 4.3.2. Variables and Data

The explained variables (production- and consumption-based carbon emissions) were calculated from the multiregional input–output table. This study used the multiregional input–output tables from 2007, 2010, 2012, 2015, and 2017, of which the multiregional input–output tables of 2007 and 2010 were prepared by Weidong Liu’ team. The multiregional input–output tables for 2012, 2015, and 2017 were obtained from China’s Carbon Emission Accounts and Datasets (CEADs). Carbon emissions data for provinces and industries were also derived from this database.

To control for the influence of other factors on the policy effect, this study introduced control variables, such as population (pop), economic development level (pgdp), urbanization rate (urban), economic export-oriented level (trade), industrial structure (industry), energy structure (coal), technical level (tec), environmental regulation intensity (evir), per capita initial industry input (industry_input), and per capita total industry output (industry_prod), which were derived from the China Energy Statistical Yearbook, China Statistical Yearbook on Environment, China Statistical Yearbook, and multiregional input–output table. Table 1 lists the descriptive statistics of the variables. It can be seen from the table that the carbon emissions of the building materials and metal industry were larger than that of the chemical industry. Whether in the chemical, building material, or metal industries, the standard deviation of carbon emissions on the production side was larger, indicating that the carbon emissions on the production side of each province were more different from the carbon emissions on the consumption side and fluctuated more.

## 5. Empirical Analysis

### 5.1. Results Based on DID Method

#### 5.1.1. Empirical Results

Table 2 and Table 3 list the evaluation results of the production- and consumption-based mitigation effects of the chemical, building materials, and metal industries, respectively. After controlling for other variables, the DID coefficients that affected the production- and consumption-based carbon emissions of the chemical industry were significant at the 5% level of −4.05 and −2.45, respectively. The DID coefficient that affected the carbon emissions on the production-based side of the building materials industry was significant at the 5% level at −6.93, while the carbon trading policy had no significant impact on the carbon emissions on the consumption side. At the 1% level, the DID coefficients affecting the production- and consumption-based carbon emissions of the metal industry were significant at −16.04 and −15.17, respectively.

Based on these results, the pilot carbon market significantly reduced the production-based carbon emissions of the chemical, building materials, and metal industries as well as the consumption-based carbon emissions of the chemical and metal industries. Overall, the pilot carbon market had a stronger inhibitory effect on carbon emissions from the production side of the chemical, building materials, and metal industries than on the consumption side, indicating that the pilot carbon market led to an increase in carbon transfer from the three industries in the pilot region to other regions (the difference between production- and consumption-based carbon emissions increased). This shows that the pilot carbon market focused more on reducing carbon emissions on the production side, leading to the outsourcing of emissions from pilot areas to non-pilot areas. Therefore, since the introduction of the carbon emissions trading scheme in the pilot area in October 2011, the carbon market effectively reduced the carbon emissions of the chemical, building materials, and metal industries in the pilot area. However, this also led to carbon leakage from these three industries from the pilot area to the non-pilot area. The degree of carbon leakage in the chemical industry was 39.4%. The degree of carbon leakage in the metal industry was weak at 5.4%. The degree of carbon leakage in the building materials industry was the highest. The pilot carbon market only reduced production-based carbon emissions but had no significant effect on consumption-based carbon emissions.

#### 5.1.2. Robustness Checks

##### Parallel-Trend Test

**Figure 2:**
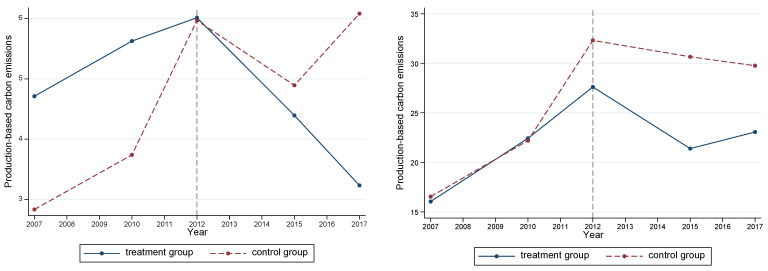

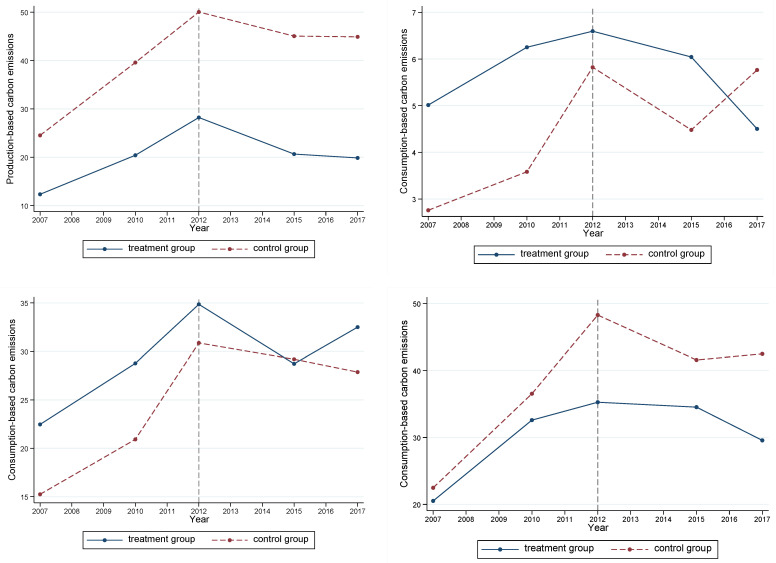
Parallel-trend test: trend over time. The first three figures are the test results of the parallel trend of production-based carbon emissions of the chemical, building materials, and metal industries, respectively. The following three figures are the test results of the parallel trend of consumption-based carbon emissions of the chemical, building materials, and metal industries, respectively.

The premise of using the DID method is that the treatment and control groups should first comply with the parallel-trend hypothesis. This indicates that the trends in the two groups before the implementation of the policy should be consistent. There are two methods to test this hypothesis: (1) draw the trend chart of carbon emissions over time in the pilot and non-pilot areas (as shown in Figure 2), and (2) test by regression. The regression model was derived as follows:

(15) $$y_{pit}=\beta_{0}+\beta_{t}\sum_{t=2007}^{t=2017}Treat\times Dyear+\beta_{1}Treat_{it}+\beta_{2}Post_{it}+\sum \beta_{j}X_{it}+\mu_{i}+\gamma +\varepsilon_{it}$$

(16) $$y_{cit}=\beta_{0}+\beta_{t}\sum_{t=2007}^{t=2017}Treat\times Dyear+\beta_{1}Treat_{it}+\beta_{2}Post_{it}+\sum \beta_{j}X_{it}+\mu_{i}+\gamma +\varepsilon_{it}$$

where *Treat × Dyear* represents the interaction item between the dummy year and dummy treatment group. If the year was before 2012, the interaction coefficient, $\beta_{t}$, between the dummy variable and dummy variable of the treatment group was not significant, indicating that there was no significant difference between the pilot and non-pilot areas before the implementation of the policy. This showed that the parallel-trend hypothesis was met.

**Figure 3:**
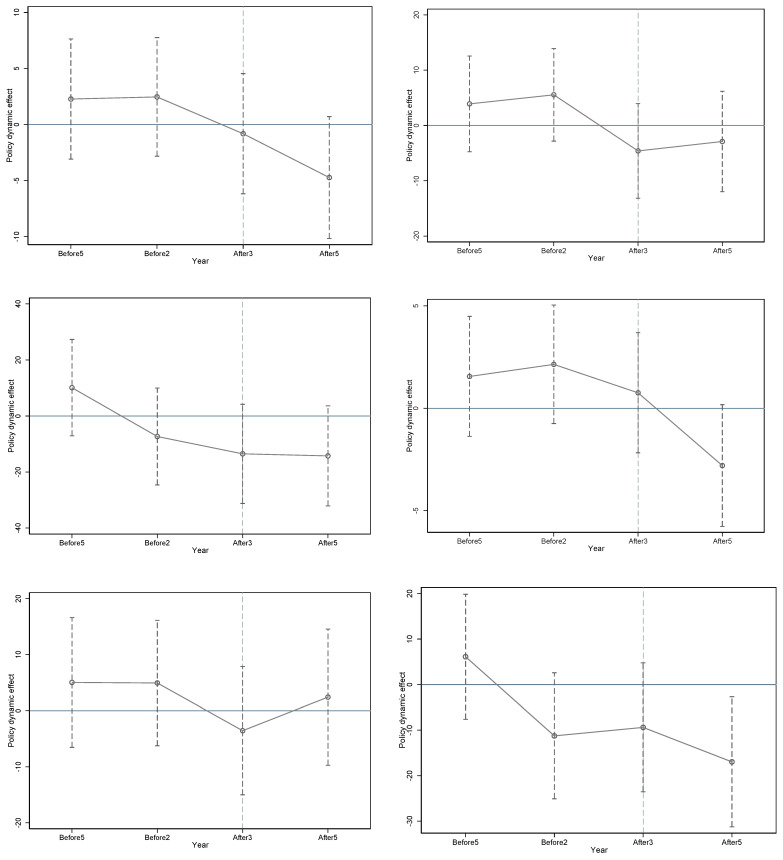
Parallel trend test: regression. The first three figures are the test results of the parallel trend of production-based carbon emissions of the chemical, building materials, and metal industries, respectively. The following three figures are the test results of the parallel trend of consumption-based carbon emissions of the chemical, building materials, and metal industries, respectively.

Based on Figure 2, before the introduction of the policy, the trends in the carbon emissions on the production and consumption sides of the three industry pilot areas and non-pilot areas over time were roughly the same. As shown in Figure 3, the interaction coefficient between the dummy variables of the three industry treatment groups and the dummy variables before 2012 was not significant. Both methods showed that the carbon emissions of the chemical, building materials, and metal industries of the treatment and control groups passed the parallel-trend hypothesis test.

##### Placebo Test

The placebo test was used to test whether after the pilot carbon market had launched, the trends of the treatment and control groups were completely caused by the carbon trading policy rather than other policies or random factors. To ensure the reliability of the empirical results, this study carried out the placebo test from two perspectives. The first was to narrow the time window, narrow the research period to 2007–2012, and change the policy implementation time. We assumed that 2010 was the year in which the carbon emissions trading system was launched. Table 4 and Table 5 list the results of the placebo test.

Based on the results, if the implementation year of the policy was advanced to 2010, the impact of the pilot carbon market on the carbon emissions (production- and consumption-based carbon emissions) of the chemical, building materials, and metal industries was not significant, indicating that the carbon emissions of these industries were hardly affected by other policies.

**Figure 4:**
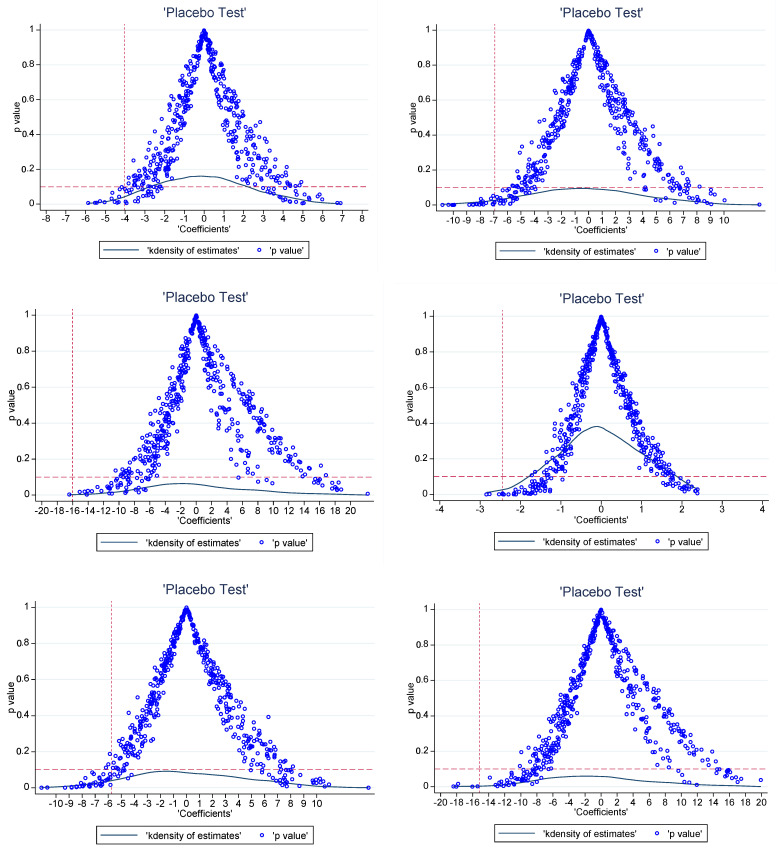
Placebo test results. The first three figures are the placebo test results of the production-based carbon emissions reduction in the chemical, building materials, and metal industries, respectively. The following three figures are the placebo test results of the consumption-based carbon emissions reduction in the chemical, building materials, and metal industries, respectively.

Second, we randomly generated the treatment groups [6]. The specific approach was to disrupt the existing treatment and control groups, randomly select six provinces (excluding pilot areas) from thirty provinces as the dummy treatment group, and list the remaining twenty-four provinces as the control group. The DID method was then used to evaluate the effect of the pilot carbon trading policy. As the randomly selected treatment group did not implement the carbon trading policy, the estimated value of the DID interaction term should be significantly different from that of the real treatment group. The placebo test process was repeated 500 times. Figure 4 shows the DID interaction term coefficients and *p*-value distribution.

Based on Figure 4, the *p*-value of most of the estimated values was greater than 0.1 (not significant at the 10% level). The real estimated value of the DID model was significantly different from most of the estimated values, which indicated that the estimated results in this study were unlikely to be obtained by chance, so the DID results of the chemical, building materials, and metal industries were unlikely to be affected by other policies or random factors.

In conclusion, a series of robustness tests showed that the DID results obtained in this study were reliable. Therefore, the pilot carbon market significantly reduced the carbon emissions on the production and consumption sides of the chemical and metal industries; the emissions reduction effect on the production side was stronger than that on the consumption side. The pilot carbon market also significantly reduced the production-based carbon emissions of the building materials industry but had no impact on consumption-based carbon emissions. The pilot carbon market caused carbon leakage from the chemical, building materials, and metal industries from the pilot area to non-pilot areas.

### 5.2. Carbon Leakage Path Analysis

We found evidence that the pilot carbon market caused carbon leakage from the chemical, building material, and metal industries from the pilot area to the non-pilot areas. To further examine the areas where the three industries in the pilot area were most likely to leak carbon, we investigated the changes in carbon transfer from the pilot area to each non-pilot area from 2007 to 2017, focusing specifically on comparative changes in carbon transfer from the pilot area to each non-pilot area before and after the pilot carbon market.

Table 6 lists the carbon emissions transferred from the pilot areas by the three major industries in each non-pilot area and the proportion of carbon emissions transferred from each province. Xinjiang, Jiangsu, Inner Mongolia, Hunan, Shandong, Henan, Hebei, Anhui, Heilongjiang, Shaanxi, Jilin and Zhejiang were the main carbon emission undertaking places of chemical products in the pilot area; the proportion of carbon emissions transfer in reached 75.95%. Henan, Hebei, Guangxi, Hunan, Jiangsu, Zhejiang, Inner Mongolia, Sichuan, Anhui, Jilin, Jiangxi and Shandong were the main carbon emissions undertaking places of building materials products in the pilot areas; the transfer in of carbon emissions accounted for more than 82.93%. Hebei, Liaoning, Jiangsu, Henan, Guangxi, Shanxi, Shandong, Yunnan, Inner Mongolia and Anhui were the main carbon emissions undertaking places of metal products in the pilot area; the transfer in of carbon emissions accounted for 78.75%.

**Figure 5:**
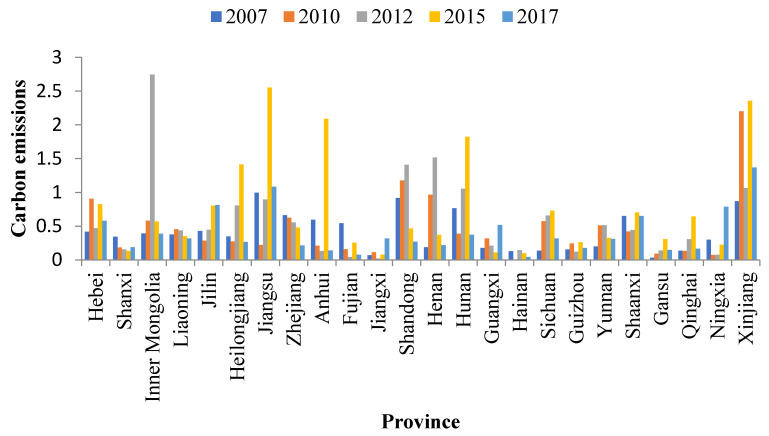
Changes in carbon transfer in the chemical industry.

**Figure 6:**
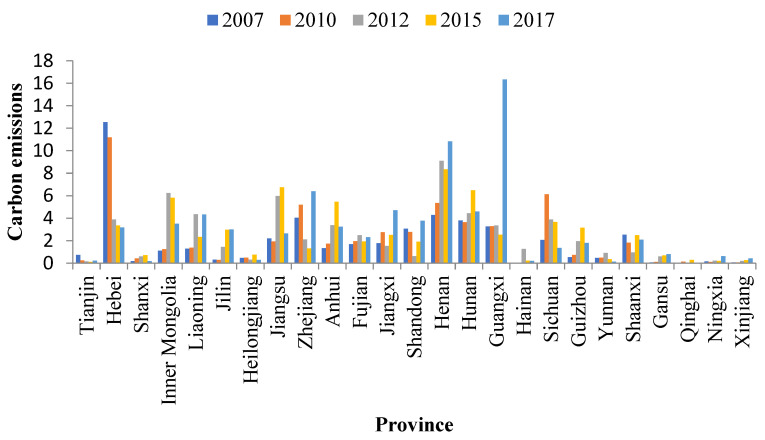
Change in carbon emissions in the building materials industry.

**Figure 7:**
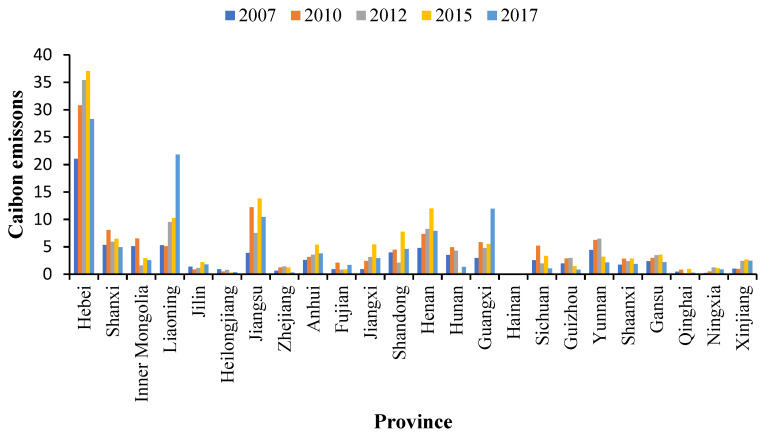
Carbon change in the metal industry.

**Figure 8:**
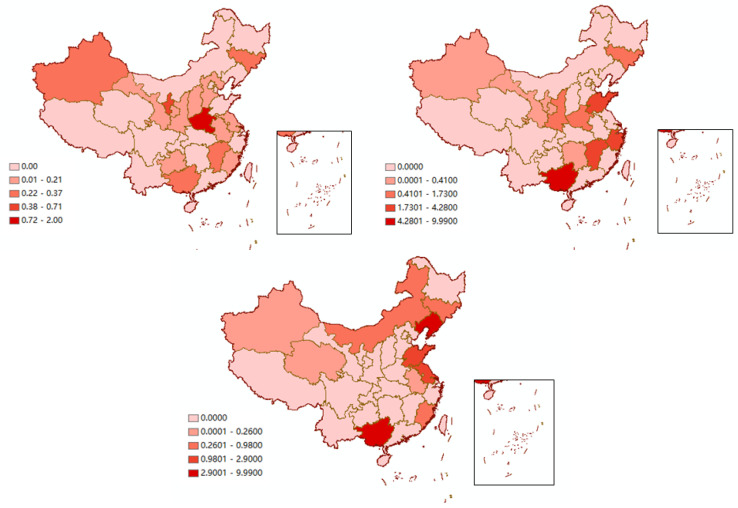
Increase in carbon transfer from pilot areas to non-pilot areas from 2012–2017 (unit: MtCO_2_). From left to right and from top to bottom: the increase in non-pilot carbon transfer from pilot areas in the chemical, building materials, and metal industries from 2012–2017.

Figure 5, Figure 6 and Figure 7 show the changes in carbon transfer from the pilot to non-pilot areas in the chemical, building materials, and metal industries from 2007–2017, respectively. Figure 8 shows the degree of carbon transfer of the three industries from pilot areas to non-pilot areas in 2012–2017. The darker the color, the more carbon transfer. As shown in Figure 5, the carbon emissions transfer from the pilot areas of the Shanxi, Jiangsu, Anhui, Fujian, Jiangxi, Guizhou, Shaanxi, and Ningxia chemical industries showed a decreasing trend from 2007–2012 and an increasing trend from 2012–2017 after the implementation of the pilot carbon trading policy. Among them, the increase in carbon emissions in Jiangsu, Anhui, Fujian, Guizhou, and Shaanxi mainly occurred from 2012–2015, with an increase of 1.66, 1.96, 0.20, 0.14, and 0.26 MtCO_2_, respectively. The carbon emissions in Shanxi mainly increased from 2015 to 2017, with an increase of 0.06 MtCO_2_. The carbon emissions transfer in Jiangxi and Ningxia increased from 2012–2015 and 2015–2017; 0.06 and 0.15 MtCO_2_ from 2012–2015 and 0.24 and 0.56 MtCO_2_ from 2015–2017, respectively, with a larger increase from 2015–2017. Additionally, the carbon emissions transfers from the pilot areas in Hebei, Jilin, Guangxi, and Xinjiang showed an increasing trend from 2007–2012 and 2012–2017, but they increased more rapidly from 2012–2017. The carbon emissions transfer in Hebei and Xinjiang increased mainly from 2012–2015, with an increase of 0.36 and 1.29 MtCO_2_, respectively. The carbon emissions transfer in Guangxi increased mainly from 2015–2017, with an increase of 0.41 MtCO_2_. The carbon emissions transfer in Jilin increased by 0.35 and 0.01 MtCO_2_ from 2012–2015 and 2015–2017, respectively. In summary, after the pilot carbon market was launched, the chemical industry in the pilot area was most likely to release carbon emissions to Shanxi, Jiangsu, Anhui, Fujian, Jiangxi, Guizhou, Shaanxi, Ningxia, Hebei, Jilin, Guangxi, and Xinjiang. Hebei, Jiangsu, Anhui, Fujian, Guizhou, Shaanxi, and Xinjiang were caused carbon leakage from 2012–2015, and Shanxi and Guangxi were caused carbon leakage from 2015–2017. Jilin, Jiangxi, and Ningxia were caused carbon leakage in two stages. Jilin was caused more carbon leakage in the first stage, whereas Jiangxi and Ningxia were caused more carbon leakage in the second stage. Particularly, Xinjiang (12.49%) and Jiangsu (9.12%) were the main carbon emissions areas with chemical products in the pilot area; therefore, there should be more of a focus on carbon leakage.

As shown in Figure 6, from 2007 to 2012, the carbon transfer of building materials from the pilot areas in industries in Zhejiang, Jiangxi, Shandong, and Shaanxi decreased. After the pilot carbon market was launched, the carbon transfer showed an upward trend. Shaanxi’s carbon transfer from the pilot areas mainly increased from 2012 to 2015, with an increase of 1.53 MtCO_2_. Zhejiang’s carbon transfer mainly increased from 2015 to 2017, with an increase of 5.07 MtCO_2_. Jiangxi’s and Shandong’s carbon transfer increased from 2012–2015 and 2015–2017, respectively. Jiangxi’s carbon transfer increased by 0.99 and 2.17 MtCO_2_ from 2012–2015 and 2015–2017, respectively. Shandong’s carbon transfer increased by 1.28 and 1.86 MtCO_2_, respectively. Additionally, carbon transfer from the pilot areas in Jilin, Guangxi, and Xinjiang showed an upward trend from 2007–2012 and 2012–2017, but it increased faster from 2012–2017. After the establishment of the pilot carbon market, carbon transfer from the pilot area in Guangxi increased from 2015–2017, with an increase of 13.81 MtCO_2_. Carbon transfer in Jilin and Xinjiang increased by 1.54 and 0.08 MtCO_2_ from 2012–2015 and 0.01 and 0.14 MtCO_2_ from 2015–2017, respectively. After the implementation of the pilot carbon trading policy, the building materials industry in the pilot area was most likely to leak carbon emissions to Zhejiang, Jiangxi, Shandong, Shaanxi, Jilin, Guangxi, and Xinjiang. Zhejiang and Guangxi were caused carbon leakage from 2015–2017, while Shaanxi was caused carbon leakage from 2012–2015. Carbon leakage in Jilin, Jiangxi, Shandong, and Xinjiang occurred in two stages. Jiangxi, Shandong, and Xinjiang transferred more carbon from the pilot areas from 2015–2017, while Jilin transferred more from 2012–2015. Particularly, Guangxi (9.49%) was the main carbon emissions area with building material products in the pilot area; these areas should avoid becoming a “pollution paradise” in the pilot area.

For the metal industry (Figure 7), carbon transfer from the pilot areas in Inner Mongolia, Jilin, Shandong, and Qinghai increased by 1.32, 1.05, 5.67, and 0.76 MtCO_2_, respectively, from 2012–2015, but decreased by 0.33, 0.42, 3.16, and 0.68 MtCO_2_ from 2015–2017. Overall, an upward trend was observed from 2012–2017. Fujian’s carbon transfer from the pilot areas increased from 2012–2015 and 2015–2017, with an increase of 0.08 and 0.81 MtCO_2_, respectively. Before the pilot carbon market was launched, carbon transfer from the pilot areas in Inner Mongolia, Jilin, Fujian, Shandong, and Qinghai showed a downward trend. Additionally, carbon transfer from the pilot areas in Liaoning and Guangxi increased by 4.21 and 1.81 MtCO_2_, respectively, from 2007–2012; carbon transfer from 2012–2017 increased more rapidly. Carbon transfer from the pilot areas in Liaoning and Guangxi increased by 0.72 and 0.74 MtCO_2_ from 2012–2015, and 11.61 and 6.40 MtCO_2_ from 2015–2017, respectively, with a larger increase from 2015–2017. Therefore, after the pilot carbon market was launched, the pilot areas were most likely to leak carbon emissions from the metal industry to Inner Mongolia, Jilin, Fujian, Shandong, Qinghai, Liaoning, and Guangxi. Inner Mongolia, Jilin, Shandong, and Qinghai were caused carbon leakage from 2012–2015, while Liaoning, Fujian, and Guangxi were caused carbon leakage from 2012–2015 and 2015–2017, but mainly from 2015–2017. Particularly, Liaoning (9.36%) was the main carbon emissions source in the pilot area undertaking metal products. These regions should focus on the increase in carbon transfer from pilot areas.

**Figure 9:**
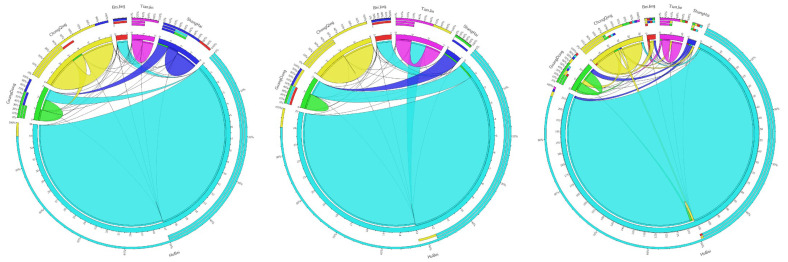

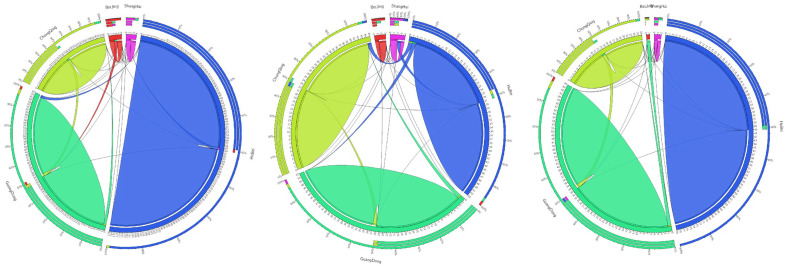

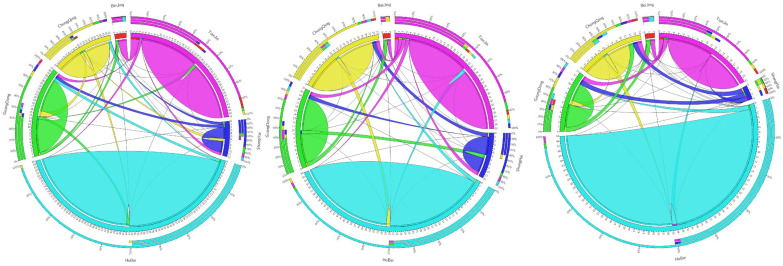
Carbon emissions flows among the pilot areas. The top three chords represent the carbon emissions flow chart between the pilot areas of the chemical industry in 2012, 2015, and 2017. The three chords in the middle represent the carbon emissions flow chart between the pilot areas of the building materials industry in 2012, 2015, and 2017. The bottom three chords show the carbon emissions flow chart between the pilot areas of the metal industry in 2012, 2015, and 2017.

Carbon leakage may occur not only between pilot areas and non-pilot areas but also between pilot areas due to different degrees of easing in terms of policy implementations (such as different carbon prices and punishment mechanisms, among others). Figure 9 shows the transfer in and out flows of carbon emissions between pilot areas in the chemical, building materials, and metal industries in 2012, 2015, and 2017. Compared with the other pilot areas, Hubei Province was a major carbon-emitting province. Whether in the chemical, building material, or metal industries, Hubei Province had the highest carbon emissions; it produced the most carbon emissions to meet its final needs. Among them, the average annual carbon emissions from the chemical industry to meet the final demand of the region was 13.01 MtCO_2_ (95.52%), that of the building materials industry was 41.27 MtCO_2_ (97.68%), and that metal industry was 33.97 MtCO_2_ (95.79%).

For the chemical industry, carbon transfer from Beijing to Guangdong; Tianjin to Guangdong; Shanghai to Guangdong; Hubei to Tianjin, Shanghai, Guangdong, and Chongqing; and Chongqing to Guangdong showed an upward trend from 2012–2017, but it showed a downward trend before the pilot carbon market was launched. Among them, the increase in carbon transfer from Beijing to Guangdong, Tianjin to Guangdong, Shanghai to Guangdong, and Chongqing to Guangdong was mainly concentrated from 2015–2017. The increase in carbon transfer from Hubei to Tianjin was mainly concentrated from 2012–2015. Carbon transfer from Hubei to Shanghai, Guangdong, and Chongqing increased from 2012–2015 and 2015–2017. Therefore, Beijing, Tianjin, Shanghai, and Chongqing were most likely to leak carbon emissions to Guangdong, while Hubei was most likely to leak its carbon emissions to Shanghai, Guangdong, and Chongqing.

In the building materials industry, carbon transfer from Beijing to Guangdong increased from 2007 to 2017. Beijing’s dependence on the building materials industry in Guangdong gradually increased: the increase from 2012–2017 was higher than that from 2007–2012. The carbon transfer from Shanghai to Guangdong, Guangdong to Hubei, and Chongqing to Guangdong showed an upward trend from 2012–2017 and a downward trend before 2012. The increase in the carbon transfer from Guangdong to Hubei and Chongqing to Guangdong was mainly concentrated from 2012–2015, while that of Shanghai to Guangdong increased from both 2012–2015 and 2015–2017. Therefore, Beijing, Shanghai, and Chongqing were most likely to leak carbon emissions to Guangdong, whereas Guangdong was most likely to leak carbon emissions to Hubei.

In terms of the metal industry, carbon transfer from Beijing to Guangdong and Chongqing to Tianjin, Shanghai, and Guangdong increased from 2007–2012 and 2012–2017, but carbon transfer from 2012–2017 increased more than that from 2007–2012. Carbon transfer from Hubei to Tianjin, Shanghai, and Guangdong and from Guangdong to Tianjin showed a downward trend from 2007–2012. From 2012–2017, these areas showed an upward trend, indicating that after the pilot carbon market implementation, Beijing was most likely to leak carbon emissions from the metal industry to Guangdong; Hubei was most likely to leak carbon emissions to Tianjin, Shanghai, and Guangdong; Guangdong was most likely to leak emissions to Tianjin; and Chongqing was most likely to leak emissions to Tianjin, Shanghai, and Guangdong. In summary, whether in the chemical, building materials, or metal industries, Guangdong was the area most likely to be caused by carbon leakage from other pilot areas.

## 6. Discussion

Previous studies by scholars have shown that there is carbon leakage from pilot areas to other areas. Different from the existing studies, this study assessed the carbon leakage in the chemical, building materials, and metal industries, specifically analyzing the regions in which the pilot regions transferred carbon emissions, providing new evidence for the existence of carbon leakage at the industry level. This paper found that the pilot carbon market caused carbon leakage in three carbon-intensive industries. Carbon leakage in the building materials industry was the most serious, followed by the chemical industry, whereas that of the metal industry was weak. This result showed that the carbon market was still more focused on reducing carbon emissions from the production side, such that enterprises in the pilot area reduced carbon emissions on the production side but transferred their carbon emissions to other regions, resulting in an increase in the carbon emissions in other regions. The degree of carbon leakage in these three industries differed. Compared with the two other industries, the metal industry had higher fixed assets and was not easily transferred; therefore, the degree of carbon leakage was weak.

We found that in the chemical, building materials, and metal industries, most of the areas affected by carbon leakage were concentrated in economically underdeveloped areas in the central and western regions, especially in the western region. Most of the relatively developed areas in the east had developed high-tech industries with low energy consumption and low emissions. Carbon-intensive industries with high emissions and pollution, such as the chemical, building materials, and metal industries, tended to be transferred to the central and western regions with relatively backward economic development, resulting in increased carbon emissions. Additionally, the eastern region had strong environmental regulations; carbon emissions in the pilot areas were transferred to the central and western regions with weak environmental regulations. Regardless of the chemical, building materials, or metal industries, after the pilot carbon market was launched, carbon transfer from the pilot areas to Jilin and Guangxi increased. Jilin is a major heavy-industry province in China, whereas Guangxi has a relatively weak ecological environment and relatively backward economic development. The phenomenon of carbon leakage caused by these two areas deserves further attention.

Regarding the carbon leakage between the pilot areas, we found that Guangdong was the area most likely to be caused by carbon leakage from other pilot areas. The policy imbalance between the pilot areas was the main reason for this result. It can be seen from Table 7 that, based on the total quota of the pilot areas, Guangdong was the most affluent province. Particularly, Guangdong still issues multiyear quotas at one time. From a carbon price perspective, compared with Beijing, Guangdong’s carbon price is relatively low. Over time, the carbon price in Guangdong has shown a downward trend. After 2015, the carbon price was less than 20 yuan/t. Simultaneously, Guangdong’s penalties for non-performing enterprises are relatively low. Overall, compared with other pilot areas, the policy environment in Guangdong in 2013–2017 is relatively loose, causing other pilot areas to transfer carbon emissions to Guangdong. Tianjin has the least-severe penalties for enterprises that fail to perform contracts on time. It prohibits enterprises that fail to comply with the contract from benefiting from preferential policies and does not provide fines. The other pilot areas are subject to specific economic penalties. This may be an important reason for the carbon emissions leakage in Hubei, Guangdong, and Chongqing from the metal industry to Tianjin.

In summary, this study further supplemented the relevant research on carbon leakage at the industry level and provided new evidence for the existence of carbon leakage at the industry level. However, this study had some limitations. On the one hand, We only considered carbon leakage from the pilot area to other non-pilot areas in China and did not examine carbon leakage to foreign countries. Theoretically, there is a possibility of carbon leakage into foreign countries. On the other hand, this study did not identify the channels through which the pilot carbon market caused carbon leakage. This study could be further optimized from two aspects. First, future studies could explore the carbon leakage from China to other parts of the world. Second, future studies could further examine the carbon leakage through carbon leakage channels and identify the specific causes of carbon leakage.

## 7. Conclusions and Policy Implications

### 7.1. Conclusions

Under the background of China’s carbon market development and efforts to promote the realization of the dual carbon target, this paper focused on the existence of carbon leakage in China’s pilot carbon market. The main conclusions of this study were as follows.

First, a carbon trading policy could promote emissions reductions. The empirical results based on the DID model showed that the carbon market significantly reduced carbon emissions on the production and consumption sides of the chemical industry, the production and consumption sides of the metal industry, and the production side of the building materials industry.

Second, the production-based emissions reduction effect of the chemical, building materials, and metal industries in the pilot areas was stronger than that of the consumption-based effects. The pilot carbon market caused the pilot areas to outsource the carbon emissions of the chemical, building materials, and metal industries to non-pilot areas, resulting in carbon leakage in the chemical, building materials, and metal industries and the aggravation of the carbon imbalance among China’s provinces. Among them, carbon leakage in the building materials industry was the most serious, followed by that of the chemical industry; carbon leakage in the metal industry was the weakest.

Third, regardless of the chemical, building material, or metal industries, most of the provinces and cities affected by carbon leakage were concentrated in regions with relatively underdeveloped economies and relatively loose environmental regulation policies, namely those in the central and western regions. Particularly, after the pilot carbon market was launched, carbon transfer from the chemical, building materials, and metal industries in the pilot area to Jilin and Guangxi increased. Jilin is a major heavy-industrial province in China. Guangxi has a relatively weak ecological environment and relatively backward economic development. The phenomenon of carbon leakage caused by these two areas deserves further attention.

Fourth, compared with other pilot areas, Guangdong was the area most likely to be caused by carbon leakage from other pilot areas. After the implementation of the pilot carbon market, the carbon emissions of Guangdong’s chemical industry from other pilot areas increased. Carbon emissions from Guangdong’s building materials industry increased from Beijing, Shanghai, and Chongqing, while carbon emissions from Guangdong’s metal industry increased from Beijing, Hubei, and Chongqing.

### 7.2. Policy Implications

This study found that the pilot carbon market caused carbon leakage, such that designs for anti-carbon leakage policies should be a major focus. If large-scale carbon leakage occurs, it will directly damage the effect of carbon market emissions reduction and affect the realization of emissions reduction goals. Based on our results and the EU’s anti-carbon leakage policy, we propose the following suggestions to manage carbon leakage.

First, the national carbon emission trading system should be improved, with priority given to industries with carbon leakage in the national carbon market to avoid carbon leakage caused by imbalances in emissions reduction policies between regions. Additionally, the EU’s anti-carbon leakage policy could be used as a reference to further optimize the initial quota allocation scheme. First, a higher proportion of free quotas should be issued to carbon-intensive industries facing carbon leakage. Second, transitional free quotas could be provided for the modernized energy sector. To prevent the adverse effects of aid measures on competition and trade, the aid intensity must be adjusted according to the actual situation.

Second, aiming to not distort market competition, we must provide appropriate assistance to enterprises in carbon-leakage industries. Particularly, we must support enterprises with upgrades and emissions reduction potential while encouraging them to improve their infrastructure and emissions reduction technologies. Relevant enterprises can submit transformation plans to government departments for government assistance, stating investments for improving infrastructure and emissions reduction technologies. Additionally, government assistance cannot fully compensate for costs incurred by enterprises. Appropriate assistance should be provided without distorting market competition. Assistance should not exceed 100% of the total investment costs listed in the plan submitted by the assisted department and must decrease over time. Otherwise, the independence of enterprises with respect to emissions reduction will decrease, which is not conducive to the independent development of enterprises. 

Third, more stringent environmental regulation policies should be formulated in areas with carbon leakage, especially in areas with fragile environmental and ecological carrying capacities. To avoid becoming a “pollution refuge”, areas affected by carbon leakage should formulate stricter environmental regulation policies. Particularly, the relevant authorities should implement the following actions: strictly review the transfer of enterprises, prevent the transfer of industries with high emissions and serious pollution, and further force pilot areas to carry out technological innovation. Environmental regulation policies and regional development strategies should be further improved based on regional heterogeneity.

## Figures and Tables

**Table 1:** Variable definitions and descriptive statistics.

Variable	Definition	Mean	Std.	Max	Min
P-emission	Production-based emissions (chemical industry)	4.722	5.395	36.704	0.072
C-emission	Consumption-based emissions (chemical industry)	4.722	3.762	18.207	0.135
P-emission	Production-based emissions (building material)	25.595	17.923	67.762	0.275
C-emission	Consumption-based emissions (building material)	25.595	15.883	79.648	1.233
P-emission	Production-based emissions (metal)	36.712	45.448	277.267	0.018
C-emission	Consumption-based emissions (metal)	36.712	26.972	126.700	0.880
pop	Population	4.505	2.752	12.141	0.552
pgdp	Per capita GDP	4.047	2.384	13.620	0.784
urban	Urban population/total population	0.543	0.136	0.915	0.282
trade	Total import and export/regional GDP	0.312	0.359	1.670	0.018
industry	Added value of secondary industry/regional GDP	0.434	0.081	0.607	0.169
coal	Coal consumption/energy consumption	0.672	0.282	1.529	0.049
tec	Total technology turnover/regional GDP	0.011	0.023	0.150	0.0002
evir	Investment in environmental pollution control/regional GDP	0.015	0.008	0.043	0.004
industry_input	Initial industry input per capita (chemical industry)	0.176	0.122	0.610	0.029
industry_prod	Total industry output per capita (chemical industry)	0.752	0.620	3.129	0.096
industry_input	Initial industry input per capita (building material)	0.075	0.053	0.277	0.007
industry_prod	Total industry output per capita (building material)	0.289	0.209	1.044	0.029
industry_input	Initial industry input per capita (metal)	0.140	0.112	0.578	0.001
industry_prod	Total industry output per capita (metal)	0.693	0.568	3.600	0.004

**Table 2:** Evaluation of production-based mitigation effects of carbon trading policy.

Variable	Chemical Industry	Variable	Building Material	Variable	Metal
DID	−4.05 ** (−2.22)	DID	−6.93 ** (−2.25)	DID	−16.04 *** (−2.61)
industry	10.65 (0.78)	pop	1.09 (0.33)	pop	10.84 * (1.70)
tec	85.5 (1.34)	pgdp	1.89 (1.34)	industry	−36.69 (−0.83)
indusry_input	24.65 * (1.71)	trade	14.88 * (1.83)	industry_prod	14.82 ** (2.27)
industry_prod	−5.39 * (−1.76)	industry	56.69 ** (2.53)	envir	−304.77 (−1.20)
				coal	−37.59 ** (−2.26)
Constant	−2.86 (−0.45)	Constant	−24.24 (−1.44)	Constant	14.93 (0.41)
Province fixed	Yes	Province fixed	Yes	Province fixed	Yes
Year fixed	Yes	Year fixed	Yes	Year fixed	Yes
Observations	150	Observations	150	Observations	150
Adjusted R^2^	0.14	Adjusted R^2^	0.58	Adjusted R^2^	0.40

Note: *, **, and *** indicate significant differences at the significance level of 10%, 5%, and 1%, respectively.

**Table 3:** Evaluation of consumption-based mitigation effects of carbon trading policy.

Variable	Chemical Industry	Variable	Building Material	Variable	Metal
DID	−2.45 ** (−2.43)	DID	−5.73 (−1.39)	DID	−15.17 *** (−3.09)
industry	8.676 (1.14)	pop	10.81 ** (2.44)	pop	13.76 *** (2.70)
tec	24.89 (0.71)	pgdp	−0.90 (−0.48)	industry	21.14 (0.60)
indusry_input	11.22 (1.41)	trade	11.20 (1.03)	industry_prod	6.48 (1.24)
industry_prod	−3.95 ** (−2.33)	industry	25.25 (0.84)	envir	66.06 (0.33)
				coal	−41.43 *** (−3.12)
Constant	−0.57 (−0.16)	Constant	44.53 ** (−1.98)	Constant	−22.33 (−0.76)
Province fixed	Yes	Province fixed	Yes	Province fixed	Yes
Year fixed	Yes	Year fixed	Yes	Year fixed	Yes
Observations	150	Observations	150	Observations	150
Adjusted R^2^	0.27	Adjusted R^2^	0.43	Adjusted R^2^	0.40

Note: ** and *** indicate significant differences at the significance level of 5% and 1%, respectively.

**Table 4:** Placebo test results of production-based carbon emissions reduction.

Variable	Chemical Industry	Variable	Building Material	Variable	Metal
DID	−0.23 (−0.14)	DID	1.38 (0.35)	DID	0.76 (0.44)
industry	14.51 (0.75)	pop	−3.17 (−0.46)	pop	−3.55 (−1.21)
tec	−60.08 (−0.71)	pgdp	−0.68 (−0.21)	industry	7.63 (0.40)
indusry_input	7.08 (0.44)	trade	7.84 (0.61)	industry_prod	4.22 * (1.75)
industry_prod	2.02 (0.52)	industry	117.69 *** (2.91)	envir	48.24 (0.74)
				coal	9.19 (1.54)
Constant	−4.65 (−0.51)	Constant	−25.34 (−0.63)	Constant	6.44 (0.37)
Province fixed	Yes	Province fixed	Yes	Province fixed	Yes
Year fixed	Yes	Year fixed	Yes	Year fixed	Yes
Observations	150	Observations	150	Observations	150
Adjusted R^2^	0.26	Adjusted R^2^	0.70	Adjusted R^2^	0.32

Note: * and *** indicate significant differences at the significance level of 10% and 1%, respectively.

**Table 5:** Placebo test results of consumption-based carbon emission reduction.

Variable	Chemical Industry	Variable	Building Material	Variable	Metal
DID	−0.23 (−0.24)	DID	2.36 (0.49)	DID	0.22 (0.22)
industry	10.99 (1.00)	pop	1.69 (0.20)	pop	−1.35 (−0.79)
tec	−8.63 (−0.18)	pgdp	−5.85 (−1.48)	industry	8.83 (0.80)
indusry_input	5.10 (0.55)	trade	6.08 (0.39)	industry_prod	1.80 (1.28)
industry_prod	0.54 (0.24)	industry	86.49 * (1.77)	envir	18.86 (0.50)
				coal	3.56 (1.02)
Constant	−2.57 (−0.50)	Constant	−20.37 (−0.42)	Constant	1.59 (0.16)
Province fixed	Yes	Province fixed	Yes	Province fixed	Yes
Year fixed	Yes	Year fixed	Yes	Year fixed	Yes
Observations	150	Observations	150	Observations	150
Adjusted R^2^	0.48	Adjusted R^2^	0.61	Adjusted R^2^	0.50

Note: * indicate significant differences at the significance level of 10%.

**Table 6:** Transfer in and proportion of carbon emissions in non-pilot areas (unit: MtCO_2_).

Province	Chemical Industry	Proportion	Building Materials	Proportion	Metal Industry	Proportion
Hebei	0.64	5.08%	6.83	11.26%	30.52	27.47%
Shanxi	0.20	1.59%	0.42	0.70%	6.15	5.54%
Inner Mongolia	0.94	7.43%	3.58	5.90%	3.75	3.38%
Liaoning	0.39	3.07%	2.74	4.51%	10.40	9.36%
Jilin	0.55	4.40%	1.61	2.65%	1.49	1.34%
Heilongjiang	0.62	4.93%	0.46	0.76%	0.59	0.53%
Jiangsu	1.15	9.12%	3.90	6.43%	9.57	8.62%
Zhejiang	0.51	4.02%	3.81	6.28%	0.98	0.89%
Anhui	0.63	5.03%	3.03	5.00%	3.71	3.34%
Fujian	0.22	1.71%	2.08	3.42%	1.29	1.16%
Jiangxi	0.12	0.95%	2.66	4.38%	2.98	2.68%
Shandong	0.85	6.72%	2.43	4.01%	4.60	4.14%
Henan	0.65	5.17%	7.58	12.49%	8.07	7.26%
Hunan	0.88	7.00%	4.59	7.56%	2.87	2.58%
Guangxi	0.27	2.11%	5.76	9.49%	6.22	5.60%
Hainan	0.08	0.67%	0.35	0.58%	0.02	0.02%
Sichuan	0.48	3.83%	3.42	5.64%	2.82	2.54%
Guizhou	0.19	1.53%	1.64	2.70%	2.03	1.83%
Yunnan	0.37	2.95%	0.47	0.78%	4.50	4.05%
Shaanxi	0.57	4.56%	1.98	3.27%	2.34	2.10%
Gansu	0.14	1.14%	0.46	0.75%	2.92	2.63%
Qinghai	0.28	2.20%	0.11	0.18%	0.56	0.50%
Ningxia	0.29	2.31%	0.27	0.45%	0.80	0.72%
Xinjiang	1.57	12.49%	0.21	0.35%	1.92	1.73%
Tianjin			0.30	0.49%		

Note: the transferred amount of carbon emissions is the average situation from 2007 to 2017.

**Table 7:** Operations of the pilot carbon market.

Pilot Areas	Time Frame	Quota Allocation Method	Average Transaction Price (yuan/ton)	Total Quota/100 Million Tons	Punishment Mechanism
Beijing	28 November 2013—31 December 2017	Historical method and baseline method	50.4	0.5	A fine of not less than 30,000 yuan but not more than 50,000 yuan shall be imposed if the reporting obligation is not fulfilled; if it fails to fulfill the obligation of quota settlement, it shall be fined not less than three times but not more than five times the average market price.
Shanghai	19 December 2013—31 December 2017	Historical method and baseline method	26.7	1.6	A fine of not less than 10,000 yuan but not more than 30,000 yuan shall be imposed for failing to fulfill the reporting obligation; a fine of not less than 50,000 yuan but not more than 100,000 yuan shall be imposed for failing to fulfill the obligation of quota settlement.
Tianjin	26 December 2013—31 December 2017	Historical method and baseline method	21.0	1.6	Within 3 years, no preferential policies related to circular economy, energy conservation, and emission reduction are allowed.
Guangdong	19 December 2013—31 December 2017	Historical method and baseline method, paid distribution of some quotas	27.1	4.2	A fine of not less than 10,000 yuan but not more than 30,000 yuan shall be imposed for failing to fulfill the reporting obligation; if the quota-clearing obligation is not fulfilled, the quota of the next year shall be deducted twice as much as the quota of the part not fully cleared, and a fine of 50,000 yuan shall be imposed.
Hubei	2 April 2014—31 December 2017	Historical method and baseline method	20.8	2.5	A fine of not less than 10,000 yuan but not more than 30,000 yuan shall be imposed for failure to fulfill the reporting obligation; if the company fails to fulfill the obligation of quota settlement, it shall impose a fine of not less than one time but not more than three times the difference according to the average market price of carbon emission quota in the current year, but not more than 150,000 yuan.
Chongqing	19 June 2014—31 December 2017	The combination of total government control and enterprise competition game	18.7	1.3	Failure to fulfill the obligation of clearing and payment of quotas shall be punished according to three times the average trading price of quotas in the month before the expiration of the clearing and payment period.

Note: work reports of China’s carbon emission trading network and pilot provincial and municipal environmental exchanges.

## Data Availability

The data used to support the findings of this study are available from the corresponding author upon request.
